# In silico identification of anti-aging pharmaceutics from community knowledge

**DOI:** 10.1093/geroni/igab046.2533

**Published:** 2021-12-17

**Authors:** Samuel Beck, Jun-Yeong Lee, Jarod Rollins

**Affiliations:** MDI Biological Laboratory, Bar Harbor, Maine, United States

## Abstract

In this era of Big Data, the volume of biological data is growing exponentially. Systematic profiling and analysis of these data will provide a new insight into biology and human health. Among diverse types of biological data, gene expression data closely mirror both the static phenotypes and the dynamic changes in biological systems. Drug-to-drug or drug-to-disease comparison of gene expression signature allows repurposing/repositioning of existing pharmaceutics to treat additional diseases that, in turn, provides a rapid and cost-effective approach for drug discovery. Thanks to technological advances, gene expression profiling by mRNA-seq became a routine tool to address all aspects of the problem in modern biological research. Here, we present how drug repositioning using published mRNA-seq data can provide unbiased and applicable pharmaco-chemical intervention strategies to human diseases and aging. In specifics, we profiled over a half-million gene expression profiling data generated from various contexts, and using this, we screened conditions that can suppress age-associated gene expression changes. As a result, our analysis identified various previously validated aging intervention strategies as positive hits. Furthermore, our analysis also predicted a novel group of chemicals that has not been studied from an aging context, and this indeed significantly extended the life span in model animals. Taken together, our data demonstrate that our community knowledge-guided in silico drug-discovery pipeline provides a useful and effective tool to identify the novel aging intervention strategy.

